# Landscape of N^6^-Methyladenosine Modification Patterns in Human Ameloblastoma

**DOI:** 10.3389/fonc.2020.556497

**Published:** 2020-10-14

**Authors:** Xing Niu, Jingping Xu, Jinwen Liu, Lijie Chen, Xue Qiao, Ming Zhong

**Affiliations:** ^1^ Department of Stomatology, Xiang’an Hospital of Xiamen University, Xiamen, China; ^2^ Department of Oral Histopathology, School and Hospital of Stomatology, China Medical University, Liaoning Province Key Laboratory of Oral Disease, Shenyang, China; ^3^ Department of Central Laboratory, School and Hospital of Stomatology, China Medical University, Liaoning Province Key Laboratory of Oral Disease, Shenyang, China

**Keywords:** m^6^A modification, ameloblastoma, messenger RNA, long noncoding RNA, circular RNA

## Abstract

**Objective:**

To comprehensively analyze the global N^6^-methyladenosine (m^6^A) modification pattern in ameloblastoma.

**Methods:**

m^6^A peaks in ameloblastoma and normal oral tissues were detected by MeRIP-seq. Differentially methylated m^6^A sites within messenger RNAs (mRNAs), long no-coding RNA (lncRNAs) and circular RNA (circRNAs) were identified, followed by functional enrichment analysis. By comprehensively analyzing MeRIP-seq and RNA-seq data, differentially expressed mRNAs, lncRNAs and circRNAs containing differentially methylated sites were identified. RNA binding proteins (RBPs) were then identified for differentially methylated m^6^A sites.

**Results:**

In total, 3,673 differentially methylated m^6^A sites within coding genes were detected, of which 16.2% (704/3,673) were significantly upmethylated sites in ameloblastoma compared to normal oral tissues. Furthermore, 4,975 differentially methylated m^6^A sites within lncRNAs were identified, of which 29.4% (1,465/4,975) were upmethylated sites in ameloblastoma. We also found 364 differentially methylated m^6^A sites within circRNAs, of which 22.5% (82/364) were upmethylated sites in ameloblastoma. Differentially methylated m^6^A was most often harbored in the CDS (54.10%), followed by 5’UTR (21.71%). Functional enrichment analysis revealed that m^6^A modification could be involved in the development of ameloblastoma by organism developmental processes. A total of 158 RBPs within differentially methylated m^6^A sites were identified, which were significantly involved in mRNA metabolic process, mRNA processing, RNA processing, RNA splicing and RNA transport.

**Conclusion:**

Our findings for the first time provide m^6^A landscape of human ameloblastoma, which expand the understanding of m^6^A modifications and uncover regulation of lncRNAs and circRNAs through m^6^A modification in ameloblastoma.

## Introduction

Ameloblastoma is the most common epithelial odontogenic neoplasm globally, accounting for approximately 36% of all odontogenic tumors (1). Ameloblastoma has a high risk of recurrence (90%) and even occurs distant metastasis (2). Surgery resection is the main treatment option for ameloblastoma, but it often leads to facial deformity, masticatory function loss, and psychological burden (3). Thus, it is of importance to develop new treatment strategies for ameloblastoma. To date, most molecular studies on ameloblastoma have focused on exploring markers and genetic variation, which help to ensure diagnosis and better determine patients’ prognosis (4–6).

m^6^A is one of the most popular RNA modifications, which is widely found in mRNA and non-coding RNA like lncRNA (7) and circRNA (8). Specific methylation of these RNA molecules regulates RNA structure and protein-RNA interactions, which may affect RNA metabolism, cell signaling, cell survival and differentiation (9, 10). m^6^A modification provides a critical transcriptomic mechanism, which regulates RNA metabolism and function. Increasing studies have confirmed that dysregulation of RNA modification like m^6^A is in association with severe human diseases including tumors (11, 12). However, no studies have reported m^6^A modification in ameloblastoma.

LncRNAs are transcribed RNA molecules with more than 200 nucleotides (13). Growing evidence suggests that lncRNAs are key regulators of gene expression, which participate in a variety of physiological and pathological processes during the occurrence and development of tumors, including ameloblastoma (14). The roles and potential mechanisms of several lncRNAs in ameloblastoma have been reported (15). The key role of m^6^A modified lncRNAs has been emphasized in cancer. For example, m^6^A modified lncRNA THOR regulates the proliferation of tumor cells (16). m^6^A reader YTHDF3 negatively mediates lncRNA GAS5 in colorectal cancer. However, the specific functions of lncRNAs with m^6^A modification remain unclear in ameloblastoma.

CircRNAs, a class of ncRNAs, have emerged as mediators of gene expression. To date, more than 100,000 circRNAs have been identified, which are expressed in specific tissues or cells and are associated with various physiological and pathological conditions (17). The biogenesis of circRNAs is regulated by various molecular factors such as RNA-binding proteins (RBPs), splicing components, proteins that affect transcriptional elongation, and the presence of reverse RNA repeats (18). CircRNAs have been shown to be functional and can affect gene expression patterns by acting as a sponge for microRNAs (miRNAs) and RBPs (18). For instance, Yang et al. found that the most abundant RNA modification motif m^6^A was enriched in the circRNA population (19). It has been demonstrated that m^6^A-modified circNSUN2 may facilitate cytoplasmic export and stabilize HMGA2, thereby accelerating colorectal liver metastasis (20). Thus, m^6^A modified circRNAs could be promising therapeutic targets for tumors. However, little is known about m^6^A modification of circRNAs in ameloblastoma. In this study, we for the first time comprehensively analyzed m^6^A modification patterns and uncovered m^6^A modified lncRNAs and circRNAs in ameloblastoma.

## Materials and Methods

### Patients and Specimens

A total of three patients with ameloblastoma were involved in our study. Tumor tissue specimens and corresponding normal oral specimens were collected during the operation from the Department of Maxillofacial Surgery, Stomatological Hospital of China Medical University. All specimens were immediately stored at −80°C before RNA isolation. Our research was approved by the Ethics Committee of School and Hospital of Stomatology, China Medical University (2019012). All participants signed written informed consents.

### RNA Preparation

Total RNA was extracted from tissue specimens using TRIzol reagent (Life Technologies, Carlsbad, CA) following the manufacturer’s instructions. RNA quantification and quality were determined using NanoDrop ND-1000 (Thermo Fisher Scientific, Waltham, MA, USA). RNA integrity and gDNA contamination were evaluated *via* denaturing agarose gel electrophoresis.

### MeRIP-Seq and RNA-Seq

MeRIP-seq and RNA-seq were performed by Cloudseq Biotech, Inc. (Shanghai, China), in accordance with a previously reported procedure (21). Briefly, m^6^A RNA immunoprecipitation was performed with the GenSeqTM m^6^A RNA IP Kit (GenSeq Inc., China) in line with the manufacturer’s instructions. Both the input sample without immunoprecipitation and the m^6^A IP samples were used for RNA-seq library generation with NEBNext^®^ Ultra II Directional RNA Library Prep Kit (New England Biolabs, Inc., USA). The library quality was evaluated with BioAnalyzer 2100 system (Agilent Technologies, Inc., USA). Library sequencing was performed on Illumina Hiseq instrument with 150bp paired-end reads. MeRIP-seq and RNA-seq data have been uploaded to the Gene Expression Omnibus (GEO) database (accession number: GSE156886).

### Data Analysis

Paired-end reads were collected from Illumina HiSeq 4000 sequencer, followed by quality control *via* Q30. Then, 3’ adaptor-trimming and low-quality reads were removed using cutadapt software (version: 1.9.3). After that, the clean reads of all libraries were aligned to the reference genome (UCSC HG19) by Hisat2 software (version: 2.0.4). CircRNAs were identified by DCC software according to STAR alignment results. Interested genes were directly visualized in the Integrative Genomics Viewer (IGV; http://www.broadinstitute.org/igv/; version: 2.4.10) (22). For MeRIP-seq, m^6^A sites on RNAs (peaks) were analyzed by overlapping three pairs of ameloblastoma and adjacent normal oral tissues using MACS software. diffReps was used to identify differentially methylated m^6^A sites between ameloblastoma tissues and adjacent normal oral tissues with the threshold of |log2 fold change (FC)|>1 and p-value<0.05. m^6^A methylation peaks that were overlapping with transcript exons were figured out and chosen by home-made scripts. For RNA-seq, raw counts were obtained based on HTSeq software (version 0.9.1), followed by normalization using the edgeR software. Differentially expressed mRNAs, lncRNAs and circRNAs were identified according to p-value and fold change.

### Functional Enrichment Analysis

The Gene ontology (GO) project provides a controlled vocabulary to describe gene and gene product attributes in any organism (http://www.geneontology.org). The ontology covers three domains: biological process (BP), cellular component (CC) and molecular function (MF). Pathway analysis is a functional analysis mapping gene to Kyoto Encyclopedia of Genes and Genomes (KEGG) pathways. In this study, functional enrichment analysis was presented for the differentially methylated mRNAs. P-value ≤ 0.05 was considered significantly enriched.

## Results

### Landscape of m^6^A Modification Patterns in Ameloblastoma

**Figure 1:**
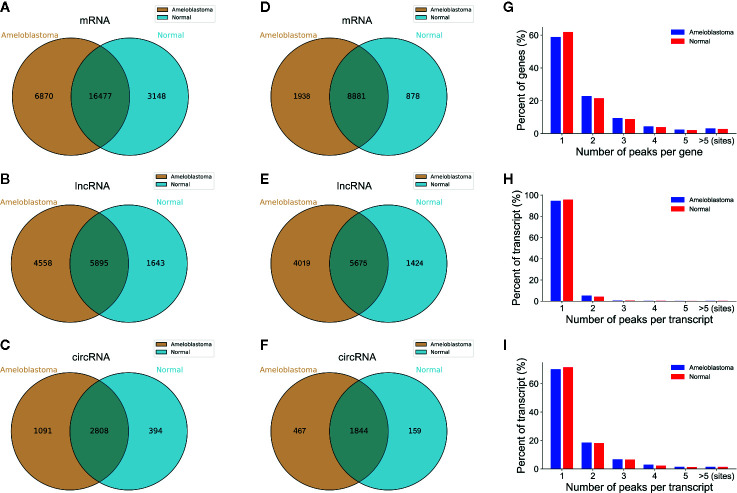
Overview of m^6^A-modified peaks within mRNAs, lncRNAs and circRNAs in ameloblastoma and adjacent normal oral tissues. **(A–C)** Venn diagram depicting the overlapped and non-overlapped m^6^A peaks within mRNAs, lncRNAs and circRNAs between the two groups. **(D–F)** Venn diagram showing the differences and overlaps in m^6^A-modified mRNAs, lncRNAs and circRNAs between the two groups. **(G-I)** The number of m^6^A peaks per mRNA, lncRNA and circRNA between the two groups.

Ameloblastoma tissues and adjacent normal oral tissues from three patients were used for MeRIP-seq analysis. Venn diagram showed that 16,477 m^6^A peaks within mRNAs (**Figure 1A**), 5,895 m^6^A peaks within lncRNAs (**Figure 1B**) and 2,808 m^6^A peaks within circRNAs (**Figure 1C**) were overlapped between ameloblastoma tissues and adjacent normal oral tissues. Noticeably, there were 6,870 nonoverlapping m^6^A peaks within mRNAs (**Figure 1A**), 4,558 nonoverlapping m^6^A peaks within lncRNAs (**Figure 1B**), and 1,091 nonoverlapping m^6^A peaks within circRNAs (**Figure 1B**) in ameloblastoma tissues compared to adjacent normal oral tissues. The high nonoverlapping percentages of m^6^A peaks within mRNAs, lncRNAs and circRNAs suggest the differences in the m^6^A modification patterns between the two groups. Furthermore, 8,881 m^6^A-modified mRNAs (**Figure 1D**), 5,675 m^6^A-modified lncRNAs (**Figure 1E**) and 1,844 m^6^A-modified circRNAs (**Figure 1E**) were found within both the two groups.

Most of m^6^A-motified mRNAs (**Figure 1G**), lncRNAs (**Figure 1H**) and circRNAs (**Figure 1I**) contained only one m^6^A peak, while a small number of them contained two or more, which was consistent with previous studies such as clear cell renal cell carcinoma (23). The top ten hypermethylated and hypomethylated m^6^A-modified peaks for ameloblastoma tissues are listed in **Tables 1**, **2**.

**Table 1:** The top ten hypermethylated m^6^A-modified peaks in ameloblastoma tissues compared to normal oral tissues.

Chromosome	txStart	txEnd	Gene name	Fold change
20	40706541	40707000	PTPRT	120.5
4	134074101	134074480	PCDH10	104.9
12	6952161	6952240	GNB3	79.8
9	87488301	87488520	NTRK2	77.6
6	70854809	70854899	COL19A1	75.1
3	49705481	49705700	BSN	73.4
1	110088121	110088320	GPR61	71.7
20	32273809	32273820	E2F1	70.7
2	136566261	136566640	LCT	68.9
1	8386101	8386102	SLC45A1	66.9

**Table 2:** The top ten hypomethylated m6A-modified peaks in ameloblastoma tissues compared to normal oral tissues.

Chromosome	txStart	txEnd	Gene name	Fold change
20	44455884	44455953	TNNC2	511.5102
14	23859601	23859655	MYH6	347.63572
1	152382941	152383240	CRNN	257.40689
11	1940841	1940992	TNNT3	232.68662
16	4292061	4292081	SRL	219.9
11	64527301	64527640	PYGM	207.14873
6	123687279	123687327	TRDN	194.63652
20	21687161	21687460	PAX1	156.8
17	10297721	10297769	MYH8	156.28189
11	62455361	62455580	LRRN4CL	154.4

**Figure 2:**
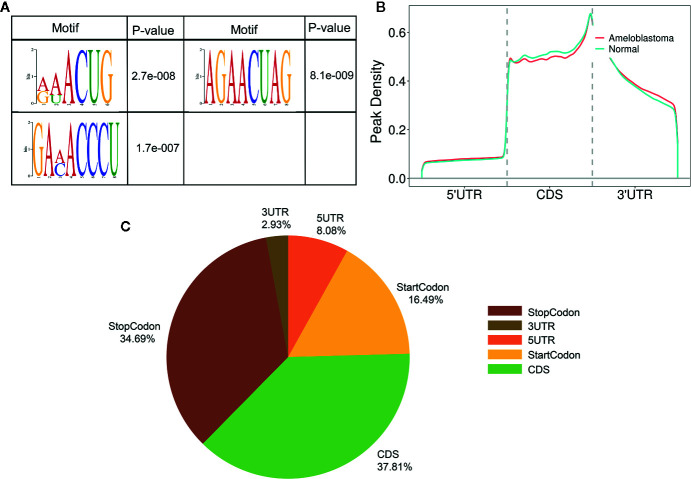
Characteristics of m^6^A peaks within mRNAs, lncRNAs and circRNAs in ameloblastoma and adjacent normal oral tissues. **(A)** Top motifs enriched from all identified m^6^A peaks within mRNAs, lncRNAs and circRNAs in ameloblastoma tissues compared to adjacent normal oral tissues. **(B)** Difference in the density of m^6^A peaks in the indicated regions between ameloblastoma and adjacent normal oral tissues. **(C)** Pie diagram showing the distributions of m^6^A peaks in the whole transcriptome of ameloblastoma tissues and adjacent normal oral tissues.

Motif analysis was performed by DREME software (version: 5.0.4) (24). Compared to adjacent normal oral samples, the top consensus motifs in the m^6^A peaks within mRNAs, lncRNAs and circRNAs were respectively AAACU, GAACU and AAACC in ameloblastoma samples (**Figure 2A**). The distribution of m^6^A was further investigated in the whole transcriptome of ameloblastoma tissues and adjacent normal oral tissues. The m^6^A peaks were mainly assigned into 5’UTR, coding sequence (CDS), start codon, stop codon and 3’UTR. As shown in **Figure 2B**, m^6^A peaks were especially enriched in the stop codon both for ameloblastoma and normal oral samples. Moreover, there was a higher peak density in the CDS region for normal oral samples compared to ameloblastoma samples. For all m^6^A peaks both in ameloblastoma and normal oral samples, the CDS was most often harbored (**Figure 2C**). The distribution of m^6^A peaks was consistent with previous m^6^A results (25).

### Abnormal m^6^A-Modified mRNAs, lncRNAs and circRNAs in Ameloblastoma

**Figure 3:**
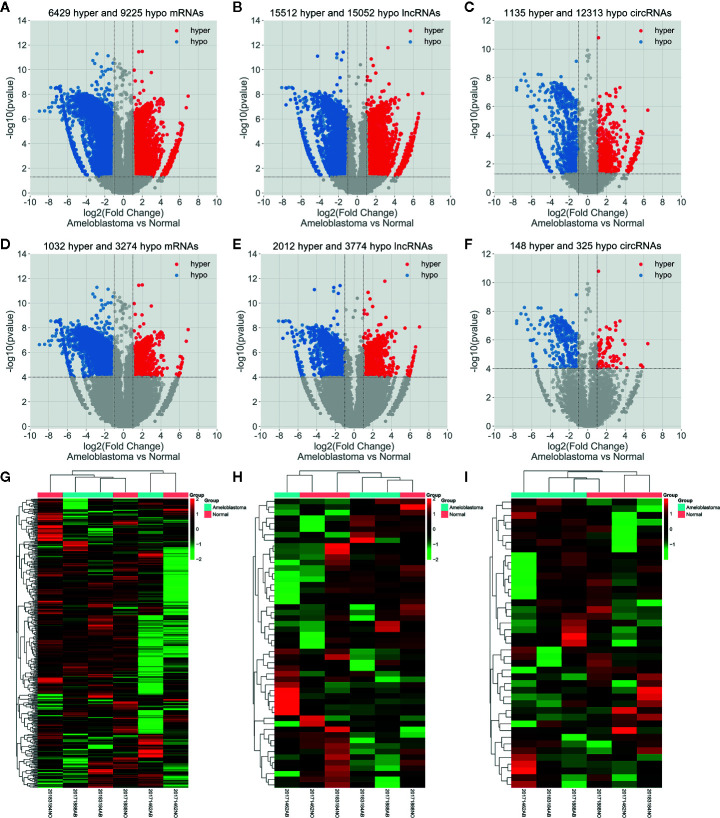
Abnormal m^6^A-modied mRNAs, lncRNAs and circRNAs in ameloblastoma tissues compared to adjacent normal oral tissues. Volcano plots showing differentially m^6^A-modified mRNAs **(A)**, lncRNAs **(B)** and circRNAs **(C)** based on |log2FC|>1 and p-value<0.05. In volcano plots, red blots represent hypermethylation and blue blots represent hypomethylation. Differentially m^6^A-modified mRNAs **(D)**, lncRNAs **(E)** and circRNAs **(F)** according to |log2 FC|>1 and p-value<0.0001. **(G–I)** Hierarchical clustering analysis results showing the differences in m^6^A modification patterns within mRNAs, lncRNAs and circRNAs between ameloblastoma tissues compared to adjacent normal oral tissues according to |log2FC|>1 and p-value<0.0001. In heat maps, red suggests hypermethylation and green suggests hypomethylation.

Abnormal m^6^A-modified mRNAs, lncRNAs and circRNAs were identified between ameloblastoma tissues and adjacent normal oral tissues. With the threshold of |log2FC|>1 and p-value<0.05, volcano plots depicted 6,429 hypermethylated and 9,225 hypomethylated mRNAs (**Figure 3A**), 15,512 hypermethylated and 15,052 hypomethylated lncRNAs (**Figure 3B**), 1,135 hypermethylated and 12,313 hypomethylated circRNAs (**Figure 3C**) in ameloblastoma tissues compared to adjacent normal oral tissues. Based on |log2FC|>1 and p-value<0.0001, we identified 1,032 hypermethylated and 3,274 hypomethylated mRNAs (**Figure 3D**), 2,012 hypermethylated and 3,774 hypomethylated lncRNAs (**Figure 3E**), 148 hypermethylated and 325 hypomethylated circRNAs (**Figure 3F**) for ameloblastoma. Then, hierarchical clustering analysis results suggested that there were significant differences in the m^6^A methylation patterns within mRNAs (**Figure 3G**), lncRNAs (**Figure 3H**) and circRNAs (**Figure 3I**) between ameloblastoma tissues and adjacent normal oral tissues.

**Figure 4:**
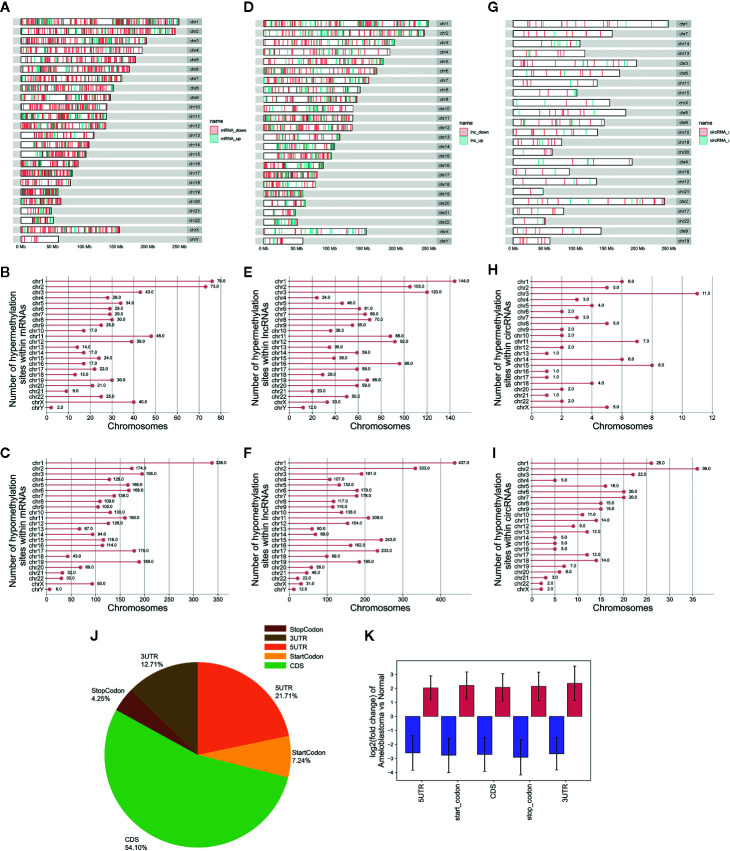
Differences in distribution of differentially methylated m^6^A sites between ameloblastoma and adjacent normal oral tissues. Chromosomal distribution of differentially methylated m^6^A sites within mRNAs **(A–C)**; lncRNAs **(D–F)**; circRNAs **(G–I)**. **(J)** Pie diagram showing the distribution of differentially methylated m^6^A sites in the whole transcriptome of ameloblastoma tissues and adjacent normal oral tissues. **(K)** The fold change of differentially methylated m^6^A sites in five regions between ameloblastoma and adjacent normal oral tissues. Red represents high methylation and purple represents low methylation.

In total, 3,673 differentially methylated m^6^A sites within mRNAs were identified, of which 16.2% (704/3,673) were significantly hypermethylated in ameloblastoma tissues compared to adjacent normal oral tissues. Furthermore, 4,975 differentially methylated m^6^A sites within lncRNAs were identified in ameloblastoma, of which 29.4% (1,465/4,975) were hypermethylated. We also identified 364 differentially methylated m^6^A sites within circRNAs, of which 22.5% (82/364) were hypermethylated in ameloblastoma. All differentially methylated m^6^A sites within mRNAs, lncRNAs and circRNAs were mapped to chromosomes to obtain their distribution profiles. The top five chromosomes harboring the most hypermethylated and hypomethylated m^6^A sites within mRNAs were 1 (76), 2 (73), 11 (48), 3 (43) and X (40); 1 (338), 3 (195), 19 (189), 17 (179) and 2 (174) in **Figures 4A–C**. The top five chromosomes harboring the most hypermethylated and hypomethylated m^6^A sites within lncRNAs were 1 (144), 3 (120), 2 (105), 16 (96) and 12 (92); 1 (437), 2 (333), 15 (243), 17 (233) and 11 (209) in **Figures 4D–F**. Moreover, the top five chromosomes harboring the most hypermethylated and hypomethylated m^6^A sites within circRNAs were 3 (11), 15 (8), 11 (7), 1 (6) and 14 (6); 2 (36), 1 (26), 3 (22), 6 (20) and 7 (20) in **Figures 4G–I**.

The distribution of differentially methylated m^6^A peaks was analyzed in ameloblastoma. In **Figure 4J**, differentially methylated m^6^A sites were most often harbored in the CDS (54.10%), followed by 5’UTR (21.71%). Then, these differentially methylated m^6^A sites were classified according to the five regions. As shown in **Figure 4K**, there were distinct differences in m^6^A levels within the five regions between ameloblastoma tissues and adjacent normal oral tissues, indicating abnormal m^6^A patterns could be involved in the development of ameloblastoma.

### Protein Coding Genes Harboring Differentially Methylated m^6^A Sites Are Involved in Important Biological Processes and Pathways

**Figure 5:**
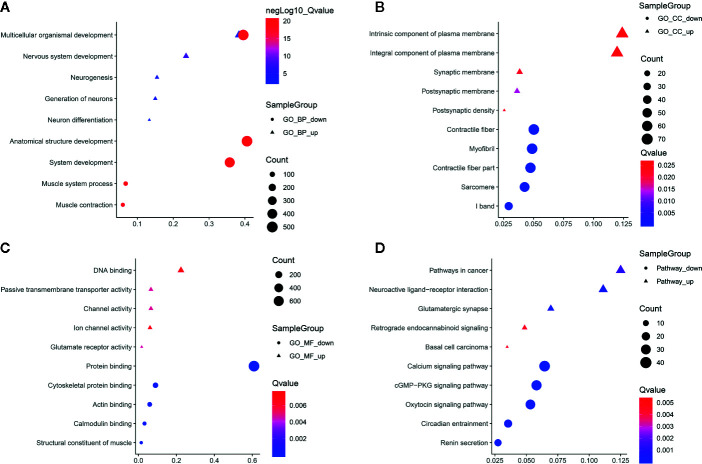
Functional enrichment analysis results of protein coding genes harboring differentially methylated m^6^A sites. The top ten enrichment results of **(A)** biological process (BP); **(B)** cellular component (CC); **(C)** molecular function (MF) and **(D)** KEGG.

To uncover the potential functions of protein coding genes harboring differentially methylated m^6^A sites in ameloblastoma, differentially methylated m^6^A sites-contained mRNAs-, lncRNAs- and circRNAs-associated genes were selected for functional enrichment analyses. For the BP term, we found that mRNAs harboring differentially methylated m^6^A sites were significantly enriched in organism developmental processes such as multicellular organismal development and system development (**Figure 5A**). For the CC term, these mRNAs harboring upmethylated m^6^A sites in ameloblastoma were mainly enriched in intrinsic/integral component of plasma membrane, postsynaptic membrane and synaptic membrane, while those harboring downmethylated m^6^A sites were highly involved in myofibril, contractile fiber and contractile fiber part (**Figure 5B**). For the MF term, these mRNAs were significantly correlated with DNA binding and protein binding (**Figure 5C**). Furthermore, these mRNAs were found to be significantly enriched in cancer-associated pathways, such as pathways in cancer, cGMP-PKG signaling pathway and oxytocin signaling pathway (**Figure 5D**).

**Figure 6:**
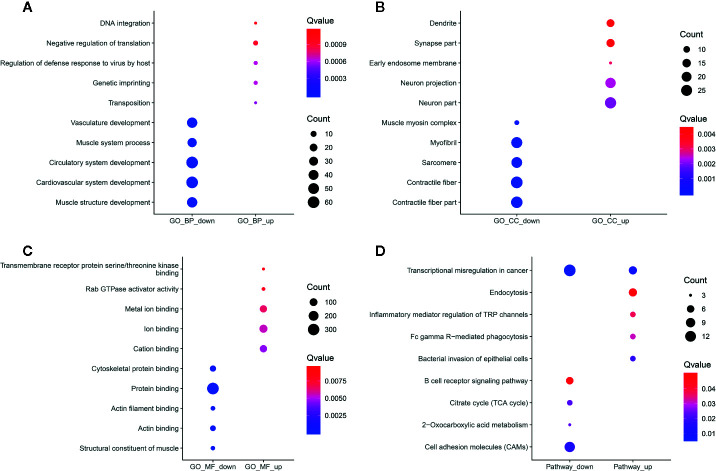
Functional enrichment analysis results of differentially methylated m^6^A sites-contained lncRNAs-associated genes. The top ten enrichment results of **(A)** biological process (BP); **(B)** cellular component (CC); **(C)** molecular function (MF) and** (D)** KEGG.

Functional enrichment analyses of differentially methylated lncRNA-associated genes were performed. For the BP category, hypermethylated or hypomethylated lncRNAs-associated genes were in a significant correlation with system development (**Figure 6A**). For the CC category, hypermethylated lncRNAs-associated genes were significantly associated with neuron part, neuron projection and early endosome membrane (**Figure 6B**), while hypomethylated lncRNAs-associated genes were mainly enriched in contractile fiber part, contractile fiber and sarcomere (**Figure 6B**). For the MF category, upmethylated lncRNAs-associated genes were mainly enriched in cation binding, ion binding and metal ion binding (**Figure 6C**), while downmethylated lncRNAs-associated genes were involved in structural constituent of muscle, protein binding and actin filament binding (**Figure 6C**). KEGG pathway analysis results showed that hypermethylated or hypomethylated lncRNAs-associated genes were significantly correlated with transcriptional misregulation in cancer (**Figure 6D**).

**Figure 7:**
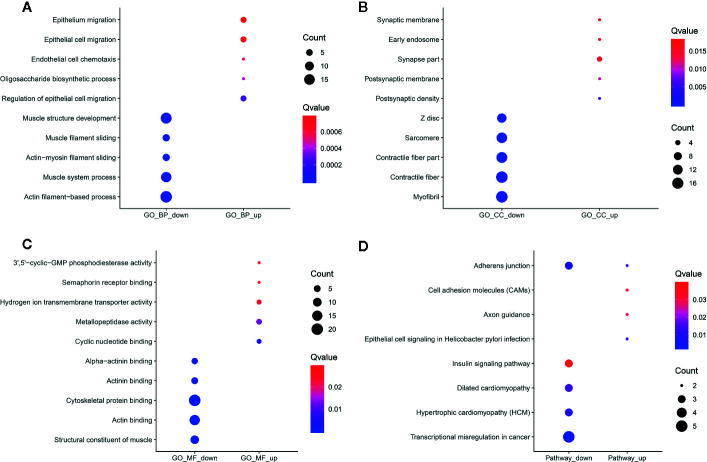
Functional enrichment analysis results of differentially methylated m^6^A circRNAs-associated genes. The top ten enrichment results of **(A)** biological process (BP); **(B)** cellular component (CC); **(C)** molecular function (MF) and **(D)** KEGG.

In **Figure 7A**, hypermethylated circRNAs-associated genes were mainly enriched in epithelial or epithelium cell migration processes, while hypomethylated circRNAs-associated genes were significantly associated with organismal processes. For the CC category, these circRNAs-associated genes were involved in postsynaptic density or membrane, synapse myofibril and contractile fiber (**Figure 7B**). For the MF category, upmethylated or downmethylated circRNAs-associated genes were mainly enriched in anion binding (**Figure 7C**). KEGG pathway analysis results showed that these circRNAs-associated genes were involved in adherens junction (**Figure 7D**).

### Identification of RNA Binding Proteins (RBPs) Within Differentially Methylated m^6^A Sites

**Figure 8:**
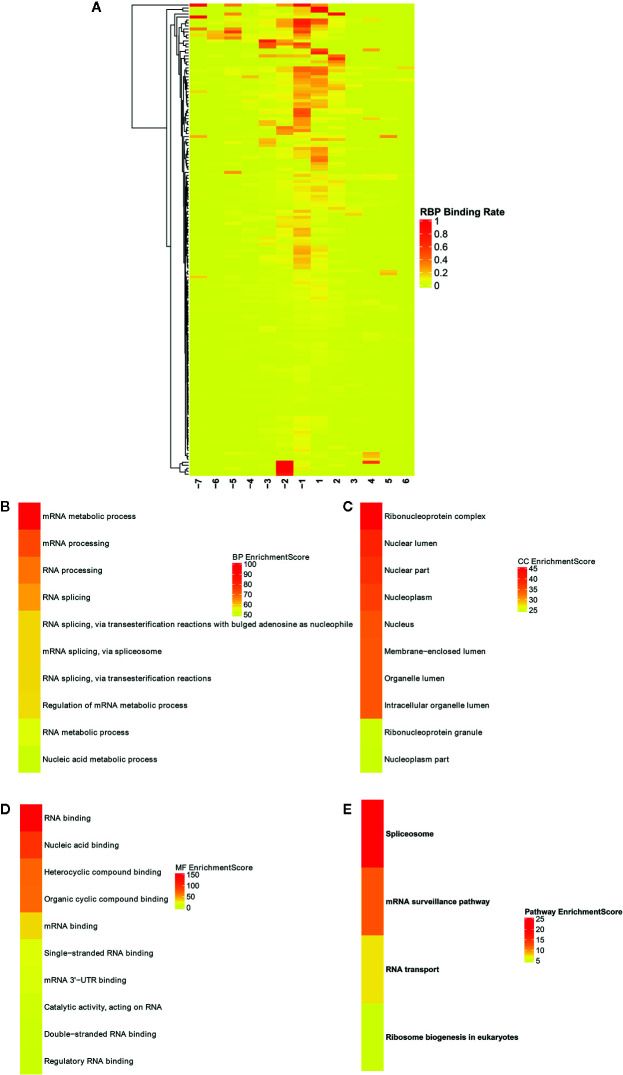
Identification of RNA binding proteins (RBPs) within differentially methylated m^6^A sites. **(A)** Heatmaps showing all 158 RBPs in ameloblastoma tissues and adjacent normal oral tissues. The top ten functional enrichment analysis results including **(B)** biological process (BP); **(C)** cellular component (CC); **(D)** membrane function (MF) and **(E)** KEGG based on these RBPs, respectively.

Potential RBPs within differentially methylated m^6^A sites were explored using RMBase database (http://rna.sysu.edu.cn/rmbase/; version: 2.0) (26). In total, 158 RBPs within differentially methylated m^6^A sites were identified, which were depicted as heat maps (**Figure 8A**). GO and KEGG enrichment analyses were then performed based on these RBPs. As shown in **Figure 8B**, for the BP term, these RBPs were mainly enriched in mRNA metabolic process, mRNA processing, RNA processing and RNA splicing. As for the CC term, these proteins were significantly associated with nuclear, nucleoplasm and ribonucleoprotein (**Figure 8C**). For the MF term, RNA binding was mainly enriched (**Figure 8D**). KEGG pathway results showed that these RBPs were significantly associated with spliceosome, mRNA surveillance pathway, RNA transport and ribosome biogenesis in eukaryotes (**Figure 8E**). These results indicated the key roles of these RBPs in regulation of gene expression, which could be involved in m^6^A modifications in ameloblastoma.

### Comprehensive Analysis of MeRIP-Seq and RNA-Seq Data in Ameloblastoma and Normal Tissues

**Figure 9:**
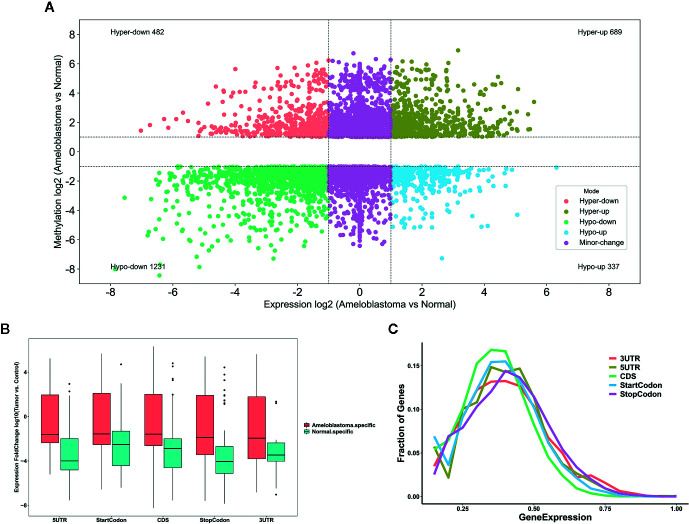
Comprehensive analysis of MeRIP-seq and RNA-seq data in ameloblastoma and normal tissues. **(A)** Scatter plot showing the distribution of mRNAs with a significant change both in m^6^A and mRNA levels between ameloblastoma tissues and normal oral tissues. **(B)** Box plot depicting the expression levels of genes in different five regions between ameloblastoma tissues and normal oral tissues. **(C)** Relative expression levels of genes in different five regions.

**Figure 10:**
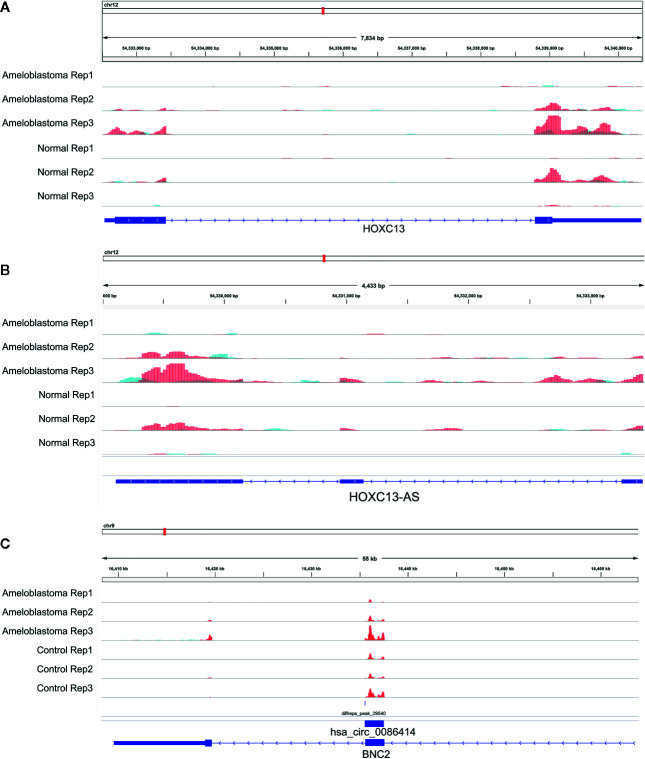
Representative differentially methylated mRNA, lncRNA and circRNA in ameloblastoma and adjacent normal oral tissues. **(A)** HOXC13; **(B)** HOXC13-AS; **(C)** hsa_circ_0086414.

We comprehensively analyzed MeRIP-seq and RNA-seq data in ameloblastoma and normal tissues. The results showed that, the expression levels of 689 hypermethylated genes tended to be up-regulated in ameloblastoma compared to normal oral tissues (**Figure 9A**). Furthermore, 482 hypermethylated genes were up-regulated in ameloblastoma, while 337 hypomethylated genes tended to be up-regulated. One thousand two hundred thirty-one hypomethylated and down-regulated genes were found, indicating that the expression levels of downmethylated genes tended to be down-regulated in ameloblastoma compared to normal oral tissues. We further analyzed the expression levels of genes containing differentially methylated m^6^A sites in five regions between ameloblastoma and normal oral tissues. As depicted in **Figure 9B**, the expression levels of these mRNAs in five regions were all higher in ameloblastoma tissues than normal oral tissues. Moreover, the CDS region had the highest fractions of mRNAs among five different regions (**Figure 9C**). In **Figures 10A–C**, we displayed the general locations of upmethylated m^6^A sites in ameloblastoma- or other oral diseases-related mRNA [HOXC13 (27)], lncRNA [HOXC13-AS (27)] and circRNA [hsa_circ_0086414 (28)] in ameloblastoma compared to adjacent normal oral tissues.

## Discussion

m^6^A is the most abundant mRNA modification in mammals. It has been well recognized that m^6^A is involved in a variety of aspects of mRNA metabolism such as mRNA translation and mRNA decay (29). Growing evidence emphasizes its important RNA biology as a hallmark of cancer (30–33). However, RNA modification in ameloblastoma is rarely reviewed. In this study, we comprehensively analyzed the differences in m^6^A modification between human ameloblastoma and normal oral tissues. We identified differentially methylated mRNAs and many important biological pathways. Furthermore, lncRNAs and circRNAs harboring m^6^A peaks were identified and these lncRNA- and circRNA-associated genes were involved in many key biological processes.

Our MeRIP-seq analysis results revealed that there were highly overlapping and non-overlapping m^6^A peaks within mRNAs, lncRNAs and circRNAs between ameloblastoma tissues and adjacent normal oral tissues. Consistent with previous studies, most of m^6^A-motified mRNAs, lncRNAs and circRNAs contained only one m^6^A peak (23). The m^6^A modification mainly occurs in the RRACH sequence (where R = A or G, H = A, C, or U). Herein, we found that the m^6^A peaks within mRNAs, lncRNAs and circRNAs were respectively enriched in the AAACU, GAACU and AAACC sequences for ameloblastoma tissues compared to normal tissues. Consistent with other tumors, m^6^A was most often enriched in the CDS and stop codon regions for ameloblastoma (34).

We identified differentially m^6^A modified mRNAs between ameloblastoma and normal oral tissues. To uncover the functions of m^6^A in ameloblastoma, we performed functional enrichment analysis of differentially methylated mRNAs. Our results revealed that mRNAs with abnormal m^6^A modification were significantly enriched in organism developmental processes, indicating that m^6^A could be involved in the development of ameloblastoma. It has been confirmed that lncRNAs play critical roles in mediating the regulation of transcription and post-transcription (35). LncRNAs are involved in chromatin organization, transcriptional, and posttranscriptional regulation (36, 37). It has been reported that lncRNA X-inactive-specific transcript (XIST) may induce the transcriptional silencing of genes on the X chromosome. As an example, RBM15 and RBM15B recruited METTL3 to methylate XIST. Furthermore, silencing RBM15, RBM15B, or METTL3 may impair XIST-mediated transcriptional repression both *in vitro* and *in vivo* (38). It has been well recognized that lncRNAs with m^6^A modification are common in human cancers (39). Up to date, only a few lncRNAs have been functionally characterized. As an example, a previous study has found that demethylated lncRNA inhibits pancreatic cancer cell motility (40). Herein, we observed the differences in m^6^A modification between human ameloblastoma and normal oral tissues, revealing a potential role for m^6^A-modified lncRNAs in the development of ameloblastoma. However, further experiments should be required to confirm these results. Although m^6^A is recognized as an abundant co-transcriptional modification in mRNAs and ncRNAs (41, 42) including circRNAs (43), it is involved in many aspects of post-transcriptional mRNA metabolism (44–46). CircRNAs exhibit patterns of m^6^A modifications that are distinct from those of mRNAs. However, little is known about the influences of m^6^A modification on circRNA biology in cells. Typically, circRNA is thought to be a co-transcript produced by canonical linear mRNA splicing that occurs in the nucleus. Herein, we identified differentially m^6^A-modified circRNAs in human ameloblastoma tissues than oral normal tissues. Recent findings have demonstrated that the export of circNSUN2 from the nucleus to the cytoplasm is dependent on m^6^A modification (20). Thus, m^6^A-modified circRNA plays a functional role in the progression of tumors, which may serve as a potential molecular marker.

RBPs act as m^6^A readers or functional factors in m^6^A modification (47). Our data identified 158 RBPs within differentially methylated m^6^A sites between ameloblastoma and normal oral tissues. As shown in functional enrichment analysis, these RBPs were mainly enriched in various processes of RNA metabolism. M^6^A modification changes the expression of target genes, thereby affecting cellular processes and physiological functions. In this study, we comprehensively analyzed MeRIP-seq and RNA-seq data in ameloblastoma and normal tissues. Our data showed that 689 hypermethylated and 482 hypermethylated genes was highly expressed in ameloblastoma compared to normal oral tissues. Furthermore, among hypomethylated genes, 337 was up-regulated and 1,231 was down-regulated in ameloblastoma tissues in comparison to normal oral tissues, indicating that m^6^A could be involved in regulation of gene expression. For example, our previous study has confirmed that HOXC13 and HOXC13-AS are both highly expressed in ameloblastoma tissues (27). Their hypermethylation was found in ameloblastoma than normal oral tissues. hsa_circ_0086414 has been detected in oral tissues (28). In this study, we found that hsa_circ_0086414 was differentially m^6^A-modified in ameloblastoma tissues in comparison to normal oral tissues. Our data indicates that m^6^A could participate in tumor progression through the modification of tumor-related genes.

## Conclusion

m^6^A modification is involved in almost every step in mRNA metabolism. Furthermore, it also affects the processing of lncRNAs and circRNAs. Our findings provide the first m^6^A modification landscape in ameloblastoma. Differentially expressed mRNAs with hyper-methylated or hypo-methylated m6A modifications are identified, which may help observe the mechanisms of m^6^A-mediated gene expression regulation. In further studies, we will evaluate the biological relevance and clinical value of m^6^A in human ameloblastoma.

## Data Availability Statement

The datasets presented in this study can be found in online repositories. The names of the repository/repositories and accession number(s) can be found in the article/supplementary material.

## Ethics Statement

The study was reviewed and approved by the Ethics Committee of School and Hospital of Stomatology, China Medical University (2019012). The patients/participants provided their written informed consent to participate in this study.

## Author Contributions

MZ conceived and designed the study. XN and JX conducted most of the experiments and data analysis, and wrote the manuscript. JL, LC, and XQ participated in collecting data and helped to draft the manuscript. All authors contributed to the article and approved the submitted version.

## Funding

This work was funded by the National Natural Science Foundation of China (81072197 and 81470758).

## Conflict of Interest

The authors declare that the research was conducted in the absence of any commercial or financial relationships that could be construed as a potential conflict of interest.

## Abbreviations

m^6^A, N^6^-methyladenosine; mRNA, messenger RNA; lncRNA, long no-coding RNA; circRNA, circular RNA; snRNA, small nuclear RNA; GO, Gene ontology; KEGG, Kyoto Encyclopedia of Genes and Genomes; RBPs, RNA binding proteins; FC, fold change.
